# Hypoxia Differentially Regulates Arterial and Venous Smooth Muscle Cell Migration

**DOI:** 10.1371/journal.pone.0138587

**Published:** 2015-09-18

**Authors:** Alice Chanakira, Devika Kir, Roderick A. Barke, Steve M. Santilli, Sundaram Ramakrishnan, Sabita Roy

**Affiliations:** 1 Department of Surgery, University of Minnesota, Minneapolis, United States of America; 2 Department of Pharmacology, University of Minnesota, Minneapolis, United States of America; 3 VA Medical Center, Minneapolis, Minnesota, United States of America; University of Nebraska Medical Center, UNITED STATES

## Abstract

**Objective:**

Intimal hyperplasia (IH) is a clinical concern leading to failure of up to 50% of vein grafts and 10% of arterial grafts after 10 years with no known current treatment. Recent studies have shown that hypoxia differentially regulates proliferation of vein derived smooth muscle cells (V-SMC) compared to artery derived smooth muscle cells (A-SMC). The objective of this study is to evaluate the effect of hypoxia on cellular migration and the mechanisms underlying the differential effects of hypoxia on A-SMC and V-SMC migration.

**Methods and Results:**

Hypoxic treatment (3–5% O2) of Smooth Muscle Cells (SMC) resulted in differential migration in scratch wound and electric cell substrate impedance sensing (ECIS) assays. Hypoxia led to greater migration compared to normoxia with venous derived wound closure (V-SMC 30.8% Normoxia to 67% Hypoxia) greater than arterial wound closure (A-SMC 6.2% Normoxia to 24.7% Hypoxia). Paracrine factors secreted by hypoxic endothelial cells induced more migration in SMC compared to factors secreted by normoxic endothelial cells. Migration of V-SMC was greater than A-SMC in the presence of paracrine factors. Neutralizing antibody to Vascular Endothelial Growth Factor Receptor -1 (VEGFR-1) completely inhibited V-SMC migration while there was only partial inhibition of A-SMC migration. A-SMC migration was completely inhibited by Platelet Derived Growth Factor BB (PDGF-BB) neutralizing antibody. p38 Mitogen Activated Protein kinase (p38 MAPK) inhibitor pre-incubation completely inhibited migration induced by paracrine factors in both A-SMC and V-SMC.

**Conclusion:**

Our study determines that SMC migration under hypoxia occurs via both an autocrine and paracrine mechanism and is dependent on Vascular Endothelial Growth Factor-A (VEGF-A) in V-SMC and PDGF-BB in A-SMC. Migration of both A-SMC and V-SMC is inhibited by p38 MAPK inhibitor. These studies suggest that pharmacotherapeutic strategies directed at modulating p38 MAPK activity can be exploited to prevent IH in vascular grafts.

## Introduction

Coronary Artery Bypass Graft (CABG) is a common surgical procedure done to treat multi-vessel or left-sided Coronary Artery Disease, mostly secondary to atherosclerosis. The most commonly used conduits to bypass these blocked arteries are the Internal Thoracic Artery (ITA), Radial Artery and the Saphenous Vein. Historically, CABG relied on the exclusive use of saphenous vein grafts until the use of ITA grafts showed improved long-term survival over a period up to 20 years compared to vein grafts alone. This was attributed to superior long-term patency rates in the ITA grafts [1–3]. In addition to ITA grafts, trials have now shown that other arterial conduits, like radial arteries, also lead to lower restenosis and better survival rates compared to saphenous vein grafts [4–8]. This restenosis or graft failure occurs because of IH, which occurs due to migration and proliferation of smooth muscle cells from the intimal layer to the medial layer in response to endothelial injury [9]. One of the factors, deemed to play an important role in inducing this response is hypoxia, which occurs during the period of graft harvesting. The graft vessels are hypoxic because of stripping away of the vasa vasorum from the adventitial surface [10]. Arterial suturing also contributes to hypoxia at the anastomosis site by hindering diffusion of blood from the luminal side [11, 12].

IH is a real clinical concern leading to failure of up to 50% of saphenous vein grafts and 10% of arterial grafts after 10 years with no known treatment so far [13]. This is due to a lack of understanding of the mechanisms responsible for IH as well as the differential response observed in arteries and veins. It has been shown that hypoxia differentially regulates proliferation of V-SMC compared to A-SMC [14]. This current study was designed to evaluate the effect of hypoxia on cellular migration and investigate if hypoxia has differential effects on A-SMC vs. V-SMC migration.

Our study shows that hypoxia differentially regulates V-SMC migration compared to A-SMC migration with VEGF-A acting through VEGFR-1 playing an important role in V-SMC vs. PDGF-BB in A-SMC. We hypothesize that this differential regulation under hypoxia explains the differences in IH observed in vein and arterial grafts.

## Materials and Methods

### Cell culture and Reagents

Human umbilical vein smooth muscle cells (V-SMC) were obtained from Science Cell Research Laboratories (Carlsbad, CA) and maintained in Smooth muscle Growth Medium-2 (SmGM-2; Lonza, Walkersville, MD). Human aortic smooth muscle cells (A-SMC) and human aortic endothelial cells (AEC) obtained from Lonza were maintained in SmGM-2 and Endothelial Growth Medium-2 (EGM-2; Lonza) respectively. Human umbilical vein endothelial cells (HUVEC) purchased from Lonza (Walkersville, MD) were a kind gift from Dr Ramakrishnan’s lab, University of Minnesota. They were cultured in EGM-2 medium (Lonza, Walkersville, MD).

Phosphorylated p38 MAPK, Total p38 MAPK primary antibodies, anti-rabbit IgG and anti-mouse IgG secondary antibodies were purchased from Cell signaling Technology (Beverly, MA). β-actin and VEGFR-1 primary antibodies were acquired from Santa Cruz Biotechnology (Santa Cruz, California); anti-human VEGFR-1 neutralizing antibody, anti-human PDGF-BB neutralizing antibody were purchased from R&D Systems, Minneapolis, MN.

SMC were serum starved for 24hr in 1% Fetal Bovine Serum (FBS) and Smooth Muscle Basal Medium -2 (SmBM-2) and then subjected to normoxia or hypoxia for different time points as indicated. Cells were exposed to hypoxia for three hours to determine changes in transcript levels. Cell migration, growth factor and growth factor receptor changes were determined after 24hr-treatment with hypoxia. For paracrine mechanism studies, endothelial conditioned media were prepared by maintaining AEC and HUVEC in 1% FBS and Endothelial Basal Medium-2 (EBM-2) and then subjecting them to normoxia or hypoxia for 24 hours. The conditioned media prepared from AEC and HUVEC were used for experiments on A-SMC and V-SMC respectively.

To achieve hypoxia (3–5% O2), cells were placed in a modular chamber (Billups Rothenberg, Inc., Del Mar, CA) and flushed with a mixture of 5% CO2, and 95% N2 at 10L/min for 15 minutes. Chambers remained tightly sealed and placed at 37°C incubator. This method achieves pO2 levels less than 35mmHg as determined from cell culture medium analyzed using a blood gas analyzer, Rapid Lab248 (Chiron Diagnostics Tarrytown, NY); pO2 levels of culture supernatant from cells grown under normoxic conditions was 150–160 mmHg.

### ECIS migration assay

To measure migration of SMC, we used the electric cell substrate impedance sensing (ECIS; Applied Biophysics, Troy, NY) that measures changes in trans cellular resistance (TER). SMCs were plated in an 8 well chamber (8W1E) and exposed to hypoxia for 24 hours. To form the wounded area, AC current was delivered to the small active electrodes located on the central part of each well to induce controlled cell death in a localized area. The wounded areas of each well were gradually healed by the migration of the viable cells surrounding the small electrodes, and the migratory response was then measured in real-time by recording the recovery of electrical impedance. For measuring the effect of paracrine factors on migration, the media in the wells were replaced with conditioned media obtained from hypoxic endothelial cells (HECM) or conditioned media from endothelial cells under normoxia (NECM), incubated with A-SMC and V-SMC and subjected to hypoxia for 24 hours. For measuring the impact of growth factors and growth factor receptors (VEGFR-1, PDGF-BB) on SMC migration, neutralizing antibodies were added to wells prior to injury and treatment with endothelial cell conditioned media.

### Scratch wound assay

SMC (50,000) were cultured in a 24-well plate until 90% confluent. SMC were serum-starved (1% FBS+ SMBM) for 24 hours. Scratch wounds were created using a 1000 μL pipette tip. Cells were treated as mentioned above. For paracrine mechanisms, SMC were treated with EBM-2, NECM or HECM after the scratch. Treatments were performed in triplicate. After 24 hours, the cells were fixed using 4% paraformaldehyde and stained with 4', 6-diamidino-2-phenylindole (DAPI). Nikon AZ100M Macro Fluorescence scope at 4x magnification was used to capture images of the scratched area to determine wound closure. Average scratch area was quantitated using NIH ImageJ software.

### Boyden chamber assay

A-SMC and V-SMC were placed on an 8-micron membrane in the upper chamber. EBM-2, NECM or HECM were placed in the bottom chamber. SMC that had not migrated were retained in the top chamber and were carefully removed by cotton swabs. SMC that migrated to the bottom side of the membrane were stained using crystal violet. After solubilization of crystal violet with DMSO, absorbance was measured at 590 nm.

### RNA isolation and cDNA preparation

RNA was extracted from SMC using 1ml TRIZOL reagent from Invitrogen (Carlsbad, CA). Total RNA (1ug) of each sample was reverse transcribed using oligo (dT) primers and RNase MMLV reverse transcriptase, according to the manufacturer’s protocol, PROMEGA (Madison, WI). cDNA (100ng) was used for RT-PCR to determine changes in transcript levels of the genes listed below. The following specific primers were used: VEGFR-1 sense: 5’-CAT CAA CCT CCC CAC CAC-3’, antisense: 5’-TAT TTT TTC AGT CCC ACA GTT AGC-3’; β-actin sense 5’-GAT CAT TGC TCC TCC TGA GC-3’ antisense 5’-CAC CTT CAC CGT TCC AGT TT- 3’. ImageJ software was used to quantify intensity of the bands obtained after running PCR product on a 1% agarose gel for gel based PCR.

### Western blot analysis (WB)

SMC were lysed with 500uL lysis buffer from Sigma (St. Louis, MO) according to manufacturer’s instructions. Total protein concentration of the supernatants was determined using Bio-Rad DC protein assay (Hercules, CA). Sixty micrograms of total protein was loaded for p38 MAPK signaling whereas fifty micrograms of protein was used for VEGFR-1 signaling. The samples were electrophoresed in a 7% discontinuous SDS-PAGE. The resolved proteins were transferred to a PVDF membrane (Bio-Rad, Hercules, CA), which was then blocked for 1 hour with 5% non-fat milk at room temperature. The membrane was incubated with (1:500) primary antibody (VEGFR-1, β actin, and p38MAPK) overnight at 4°C. Membranes were then treated with anti-mouse or rabbit IgG secondary antibodies (1:1000) conjugated with horseradish peroxidase. Immunoblots were detected with UltraQuant 6.0 Ultralum (Claremont, CA) using enhanced chemiluminescence technique (Immobilon Western HRP Substrate; Millipore) (Billerica, MA). Quantification of bands was performed using UltraQuant 6.0 (Claremont, CA).

### Statistics

All experiments were done at least in triplicates and repeated three times independently. Data are expressed as means ± SD. Differences in mean values between the two groups were analyzed using the two-tailed Student’s t-test. *P* < 0.05 was considered statistically significant.

## Results

### Venous smooth muscle cells migrate faster under hypoxia than arterial smooth muscle cells

**Fig 1:**
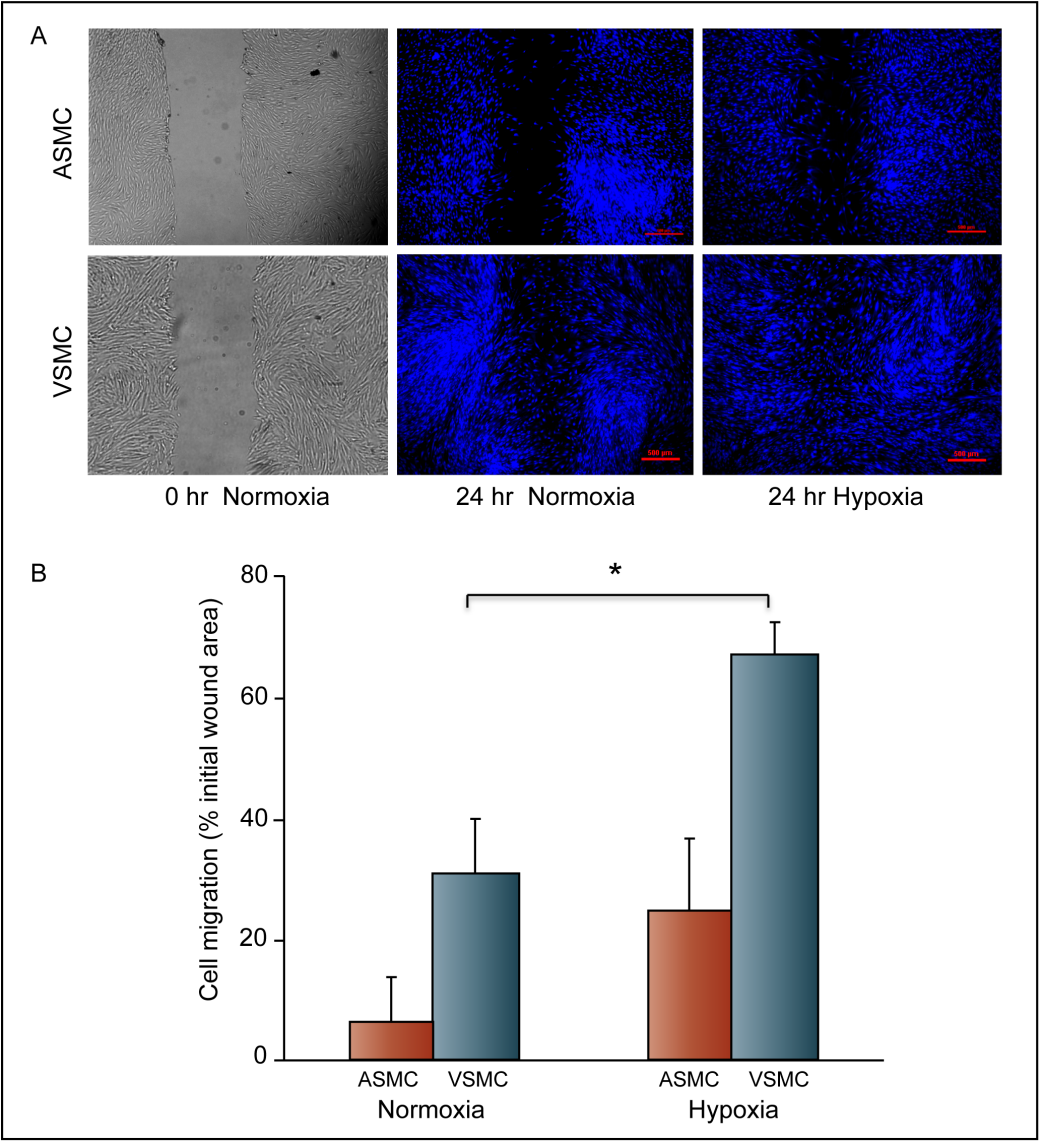
Hypoxia induced migration of V-SMC and A-SMC. Scratch wound assays were used to determine relative migration of cells under normoxia or hypoxia. **A.** Representative phase contrast images of V-SMC and A-SMC immediately after the scratch (0 hour) are shown. Cells were fixed and stained with DAPI after 24-hour treatment with normoxia or hypoxia. Representative images are shown. **B.** Relative migration and wound closure was determined in three independent experiments. Error bars indicate S.D. Data are the mean ± SD of at least three independent experiments. * represents *P*<0.05.

**Fig 2:**
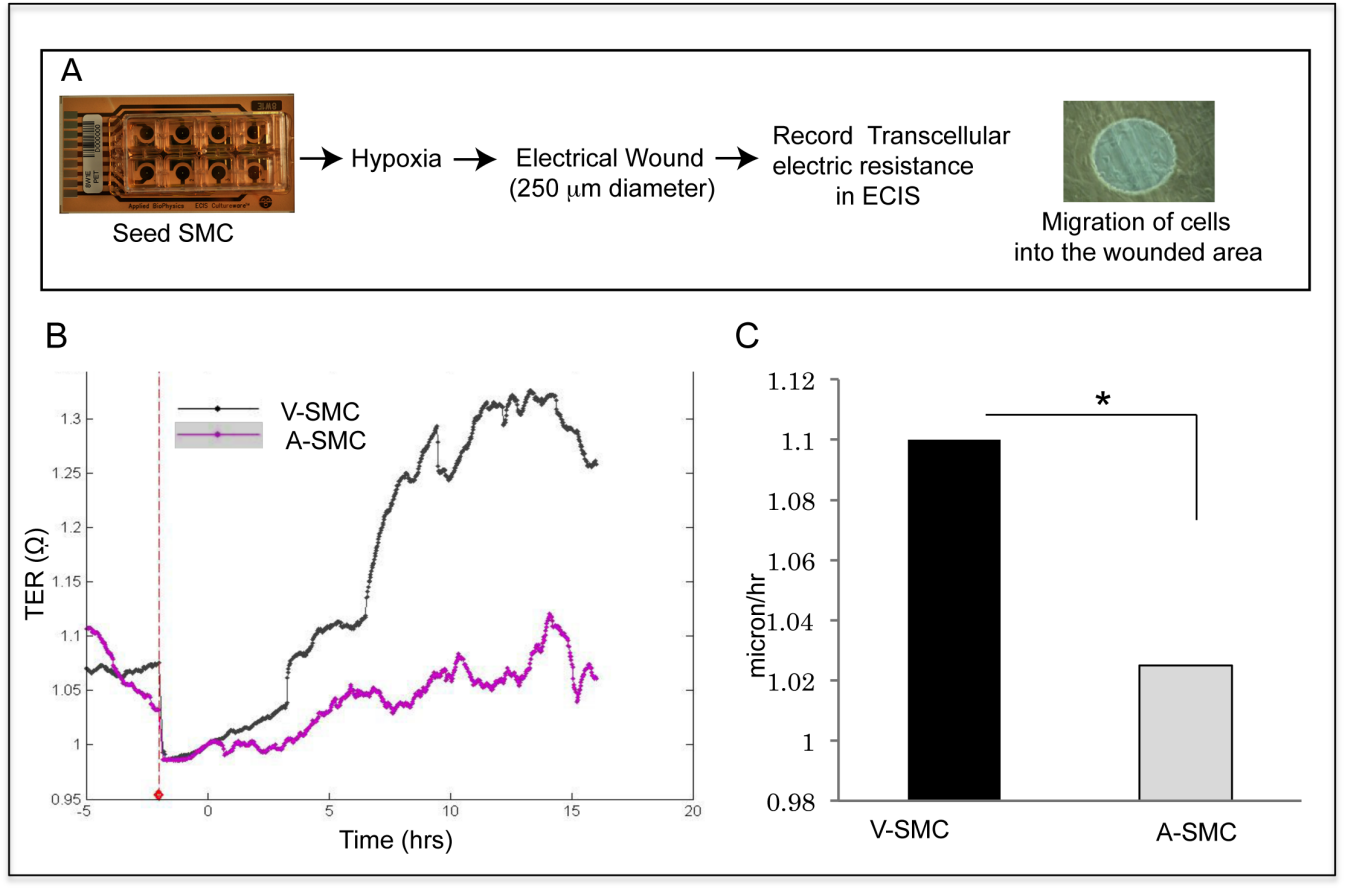
Real-time cell migration. ECIS was used to determine TER. **A.** Flow chart of the experimental design. **B.** Real-time tracings of V-SMC and A-SMC, normalized to the time of wounding, representing average cellular migration over a period of 15 hours are shown. The tracings represent average TER (quadruplicates) of V-SMC and A-SMC. **C.** Histogram shows average rate of SMC migration from quadruplicate cultures over 5 hours. * indicates statistical significance (*P* <0.05).

Scratch wound assays were carried out to determine the effect of hypoxia on A-SMC and V-SMC migration. Confluent cultures were subjected to scratch assay and then placed under normoxia or hypoxia for 24 hours. Cells were stained with DAPI and wound closure was determined using NIH ImageJ software. V-SMC showed higher basal migration than A-SMC. Data in Fig 1A shows representative images of the scratch wound assay. Fig 1B summarizes mean migration of cells from three independent experiments. Under normoxic conditions, A-SMC showed 6.2% of closure of the wound area in 24 hours while V-SMC showed a wound closure of 30.8% under the same conditions. A-SMC showed a four-fold increase (24.7% wound closure) in migration under hypoxic condition when compared to normoxia. V-SMC responded to hypoxia and narrowed the wound area more efficiently, closing 67% of the original wound area. These studies show that hypoxia differentially affects A-SMC and V-SMC migration. While hypoxia stimulated both A-SMC and V-SMC to migrate more when compared to normoxia, overall migration of V-SMC was significantly greater than A-SMC (Fig 1B). To further confirm the differential effects of hypoxia on SMC migration the following experiments were performed using real-time cell monitoring of cellular migration using trans cellular resistance measurements. A schematic diagram summarizing the assay format is illustrated in Fig 2A. Real-time recording of cell movement into the wounded area is shown in Fig 2B. Cell migration was traced over a period of 15 hours. The slope of the tracings showed that V-SMC migrated at a faster speed of about 1.1 micron/hour when compared to A-SMC, which migrated at a speed of 1.02 micron/hour. Fig 2C shows histogram of cell migration after 5 hours. These data suggest that basal migration of V-SMC is higher than A-SMC and that V-SMC migration is further stimulated by hypoxia-induced autocrine mechanisms to a greater extent than A-SMC.

### Paracrine stimulation of Smooth Muscle Cells by endothelial cells exposed to hypoxia

**Fig 3:**
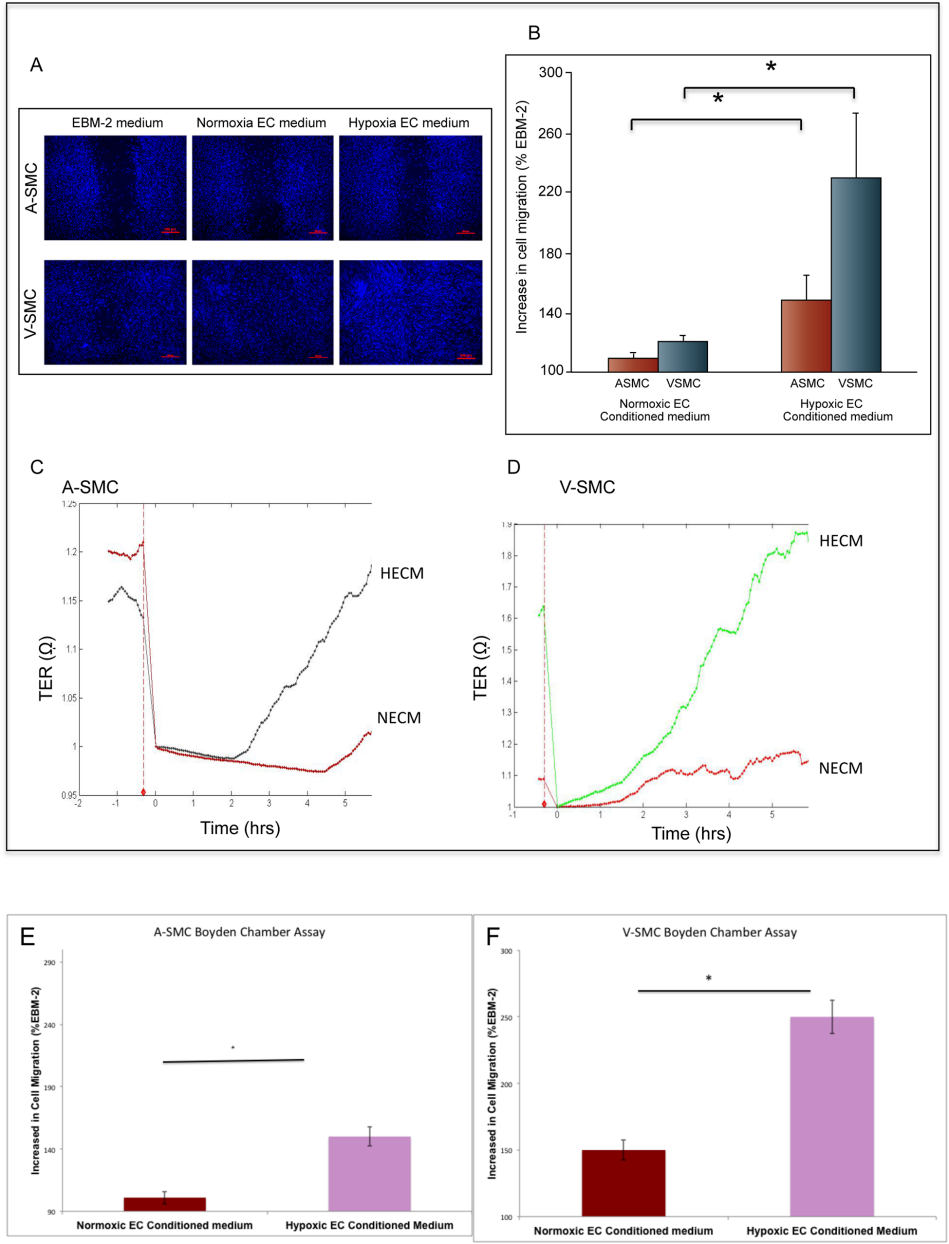
Hypoxic endothelial cell conditioned medium augments SMC migration. **A. and B.** Trans cellular electrical resistance (TER) tracing indicates real-time cell migration. Endothelial cell-derived growth factors in HECM stimulated SMC to migrate faster when compared to NECM. **C.** Effect of endothelial cell conditioned media (NECM and HECM) on SMC migration in scratch wound assays. Cell migration was normalized to EBM-2 control (100%). Error bars indicate S.D. Data are the mean ± SD from three independent experiments. * Represents *P*<0.05. D and E. Effect of endothelial cell conditioned media (NECM and HECM) on SMC migration in Boyden chamber assays. Values are normalized to EBM-2 control (100%). Error bars indicate S.D. Data are the mean ± SD of three independent experiments.

After establishing the differential response of SMC from arteries and vein to hypoxia, investigations were carried out to determine whether endothelial cells could regulate SMC migration under hypoxia. AEC and HUVEC were cultured in low serum EBM-2 medium for 24 hours either in normoxia or hypoxia. Effects of endothelial cell conditioned media on migration of A-SMC and V-SMC in scratch wound assays were studied. Data in Fig 3A shows representative images of the scratch wound assay. Fig 3B shows the effect of endothelial cell- derived paracrine factors on SMC migration from three independent experiments. Basal migration in EBM-2 medium was considered as 100% in these studies. Conditioned media from endothelial cells grown in normoxia (NECM) showed marginal effect on A-SMC migration. V-SMC migrated more (120%) compared to A-SMC under similar conditions. Endothelial cells cultured in hypoxia however stimulated SMC to migrate more. When stimulated by paracrine factors in HECM, A-SMC showed 140% higher migration while V-SMC showed 220% higher migration over the control basal medium, i.e., EBM-2. These results were further confirmed in real-time migration assays by impedance measurements. Fig 3C and 3D show the relative rate of migration of V-SMC and A-SMC in the presence of NECM and HECM. Hypoxia treated endothelial cells secreted factors into the culture media that stimulated greater migration in SMC. The tracings shown in Fig 3C and 3D represent cellular migration over a period of 5 hours. The slope of the tracings showed that V-SMC migrated at a higher rate upon addition of HECM when compared to NECM. These results were further confirmed in Boyden chamber assays. In this assay EBM-2, NECM or HECM were placed in the bottom chamber of the well and SMC placed on top of an 8uM membrane. Cells that migrated across the membrane were stained with crystal violet, washed, solubilized by DMSO and absorption was measured. Fig 3E and 3F show the effect of NECM and HECM on SMC migration. Boyden chamber assay again confirmed that endothelial cells secrete paracrine factors and stimulate SMC migration. V-SMC cells are more sensitive to factors secreted by endothelial cells exposed to hypoxia. Previously it has been shown that VEGF-A and PDGF-BB are the two main growth factors greatly induced under hypoxia in endothelial cells [14]. VEGF-A is known to induce smooth muscle cell migration through VEGFR-1 dependent pathway [15]. To determine if VEGF-A and PDGF-BB actually play a role in the differential effects of hypoxia on SMC migration the following studies were conducted.

### Antibodies to VEGFR-1 and PDGF-BB inhibit stimulation of SMC migration by endothelial cell- derived growth factors

**Fig 4:**
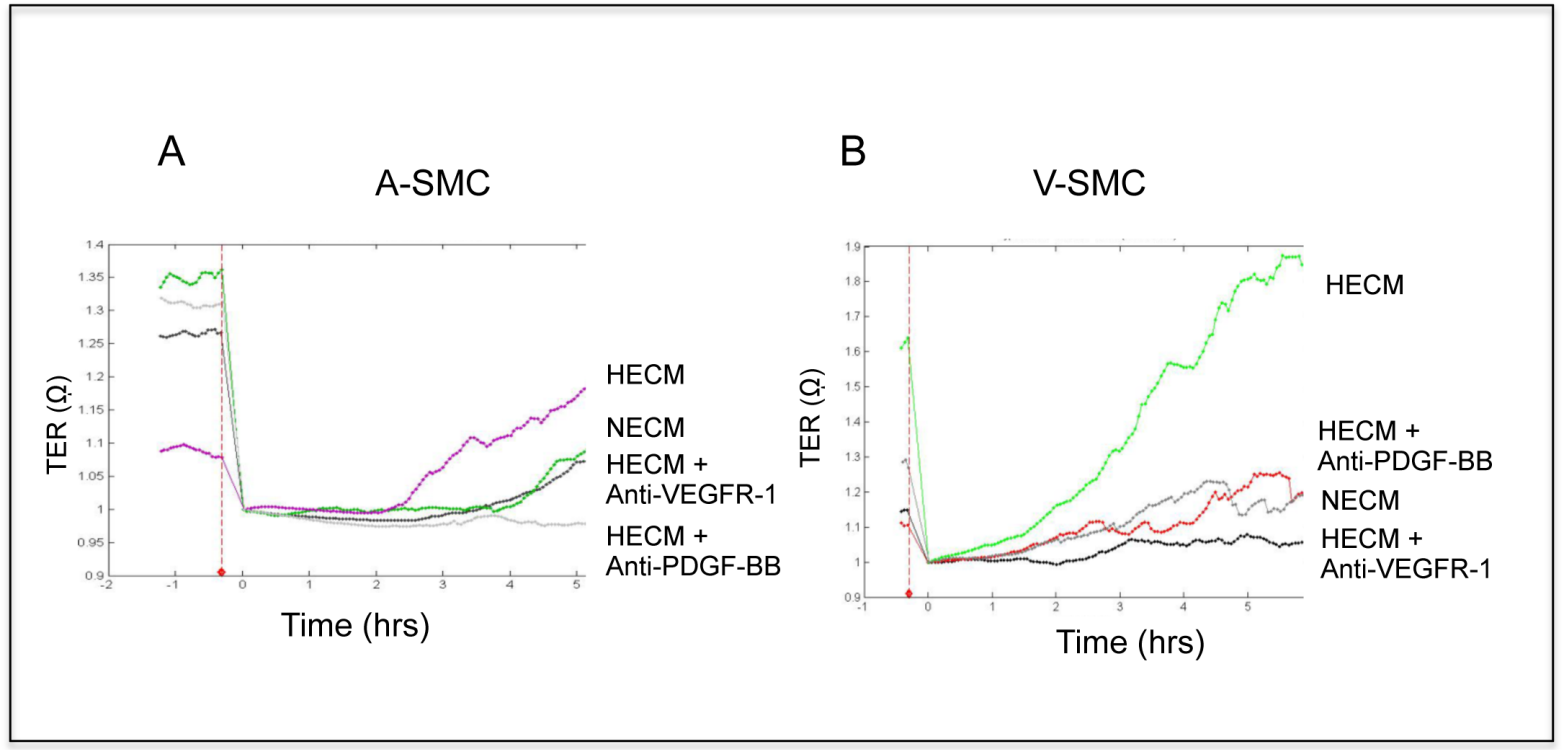
VEGFR-1 and PDGF-BB play a role in SMC migration. **A. and B.** Real-time tracings of A-SMC and V-SMC, normalized to the time of wounding, representing average cellular migration over a period of 5 hours, with or without pre-incubation with VEGFR-1 and PDGF-BB neutralizing antibody, in the presence of HECM are shown.

Electrically wounded A-SMC and V-SMC cultures were treated with VEGFR-1 neutralizing antibodies prior to the addition of endothelial cell- conditioned media obtained from normoxic and hypoxic cultures. Upon pre-incubation with VEGFR-1 neutralizing antibody there was complete neutralization of V-SMC migration stimulated by HECM. Under similar conditions, neutralization of PDGF-BB in the conditioned medium showed partial inhibition of V-SMC cell migration (Fig 4B). These results suggest that V-SMC respond to VEGF-A and PDGF-BB secreted by hypoxic endothelial cells. Interestingly, A-SMC tracings showed complete neutralization of migration of A-SMC by PDGF-BB antibody (Fig 4A) in contrast to the partial neutralization of V-SMC migration. Furthermore, VEGFR-1 antibody showed only a partial decrease in A-SMC migration in the presence of HECM. These studies suggest that VEGF-A mediated signaling through VEGFR-1 is more important for V-SMC migration compared to A-SMC migration. On the other hand, A-SMC migration seems to be modulated by PDGF-BB secreted by hypoxic AEC.

### Relative expression of VEGFR-1 by V-SMC and A-SMC under hypoxia

**Fig 5:**
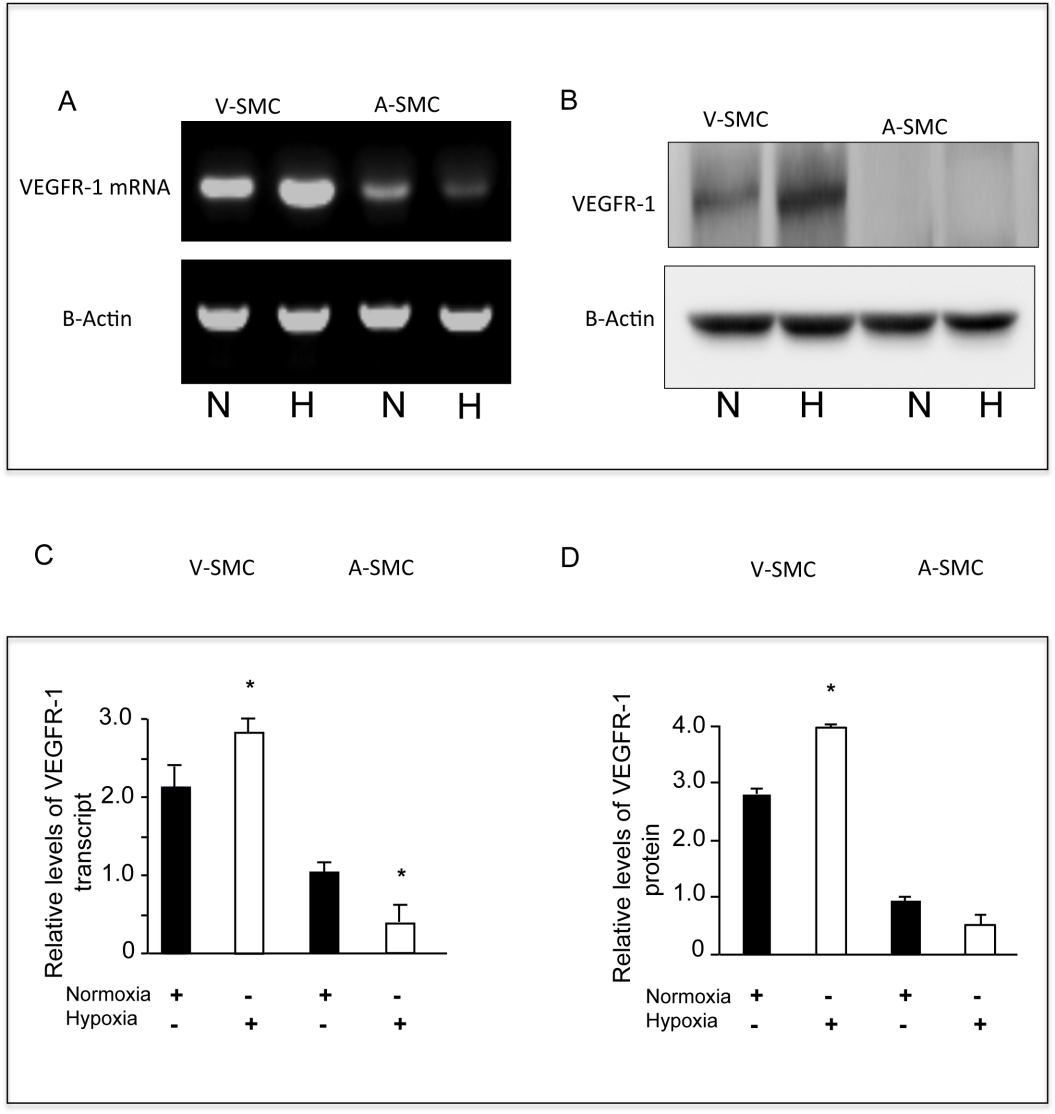
Hypoxia differentially up regulates VEGFR-1 in SMC. **A.** RT-PCR was used to determine hypoxia-induced changes in VEGFR-1 transcript levels. Representative gel showing VEGFR-1 mRNA levels in V-SMC and A-SMC under normoxia and hypoxia. **B.** Representative western blot showing hypoxia induced changes in VEGFR-1 protein levels in V-SMC and A-SMC. **C.** RT-PCR amplified fragments were resolved in agarose gels and scanned. Values from three independent experiments are shown. Transcript levels in A-SMC under normoxic conditions are considered as 1 to calculate relative transcript levels in V-SMC in normoxia and hypoxia. Error bars indicate S.D. * Represents *P* <0.05. **D.** The figure represents relative levels of VEGFR-1 protein, normalized to A-SMC levels under normoxia. Error bars indicate S.D. Data are the mean ± SD of at least three independent experiments. * represents *P* <0.05.

In order to better understand the differential response to VEGFR-1 inhibition in AMSC and V-SMC, we determined relative transcript levels of VEGFR-1 in V-SMC and A-SMC grown under normoxia or hypoxia. RT-PCR studies showed that V-SMC have higher basal levels of VEGFR-1 when compared to A-SMC (Fig 5A). When V-SMC were cultured under hypoxia there was a 1.4-fold induction in VEGFR-1 message. On the contrary, A-SMC showed a 0.5-fold reduction in VEGFR-1 transcripts when compared to normoxia (Fig 5C). These results were then confirmed by western blot. Again, V-SMC expressed higher levels of VEGFR-1 than A-SMC even under normoxic conditions (Fig 5B). Data from three independent western blots are summarized in Fig 5D. When message levels were normalized to A-SMC (normoxia), V-SMC showed 2.8-fold induction of VEGFR-1 under basal conditions. Hypoxia further increased VEGFR-1 levels to about 4-fold. Under similar conditions, A-SMC showed moderate induction in VEGFR-1 levels under hypoxic conditions. (Fig 5D).

### Endothelial cell secreted paracrine growth factors signal through p38 MAPK pathway

**Fig 6:**
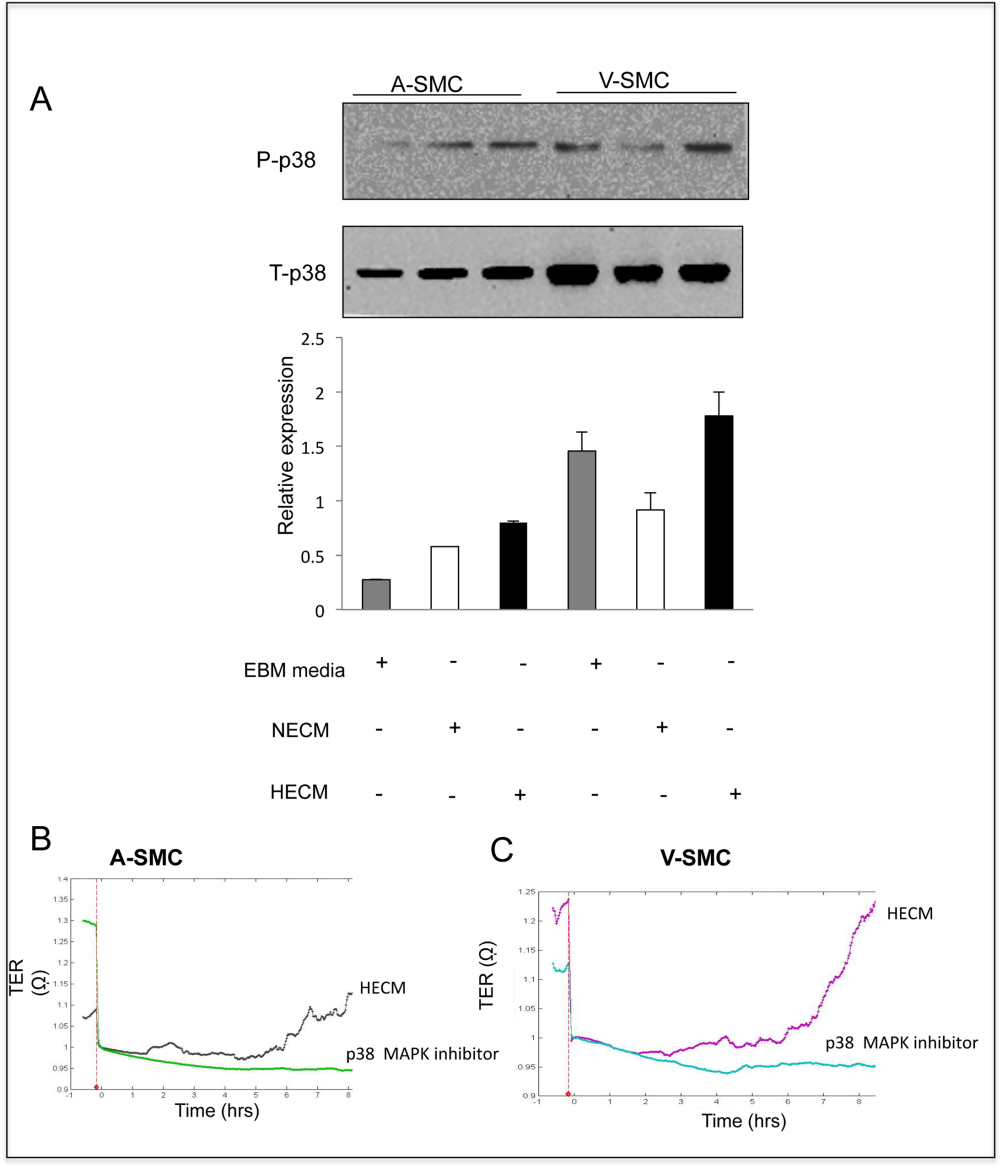
p38 MAPK pathway is involved in the paracrine-stimulation of SMC by endothelial cells. **A.** Upper panel: Representative western blot shows the levels of phosphorylated p38 MAPK and total p38 MAPK in A-SMC and V-SMC, when incubated with EBM-2, NECM and HECM under hypoxia for 3 hours. Lower panel: The figure represents relative levels of phosphorylated p38 MAPK normalized to total p38 MAPK levels. Error bars indicate S.D. Data are the mean ± SD of at least three independent experiments. **B. and C.** Real-time tracings of V-SMC and A-SMC, normalized to the time of wounding, representing average cellular migration with or without SB203580 (p38MAPK inhibitor), in the presence of HECM, over a period of 8 hours. The tracings represent average TER (quadruplicates) of V-SMC and A-SMC in the presence or absence of SB203580.

p38 MAPK has been implicated in SMC migration in response to growth factors [16, 17]. V-SMC and A-SMC were treated with NECM or HECM and then subjected to hypoxia for 3 hours. The lysates were collected and total and phosphorylated p38 MAPK protein levels were determined using western blots. Our data shows an increase in phosphorylated p38 MAPK levels in both A-SMC and V-SMC upon addition of HECM. However, V-SMC showed a greater p38 MAPK induction compared to A-SMC (Fig 6A). Next, we investigated the effect of SB203580, a well-known p38 MAPK inhibitor, on V-SMC and A-SMC migration stimulated by HECM. Real-time cell migration studies showed that SB203580 (p38 MAPK inhibitor) completely inhibited migration of both V-SMC and A-SMC induced by HECM (Fig 6B and 6C). These studies suggest that paracrine factors secreted by endothelial cells exposed to hypoxia induce SMC migration by activating p38 MAPK pathway.

## Discussion

Coronary Artery Disease (CAD) was the underlying cause in 1 of every 7 deaths in the US in 2011 [18]. Long-term treatment modalities for CAD include Percutaneous Intervention (PCI) in the form of Drug-eluting stents and CABG. Recently, a randomized controlled trial (BEST) comparing Everolimus-eluting stents (PCI) with CABG concluded that CABG was superior to PCI at a long-term follow-up of 4.6 years. The primary end-point in this trial was a composite of death, myocardial infarction or target-vessel revascularization [19]. However, CABG too suffers from the issue of restenosis at the anastomosis site due to IH. Over a period of 10 years after bypass, about 50% of saphenous vein and 10% of arterial grafts fail due to restenosis [13]. Despite intensive research, there is no effective pharmacotherapy to prevent graft failure as yet. This may be due to incomplete understanding of the mechanisms leading to graft failure, especially in the saphenous vein grafts. Some of the hemodynamic factors deemed important for causing IH in the vein grafts are high pressure, high shear stress, pulsatile flow in the arterial circulation and difference in elastic properties between the vein graft and the host vessel [20]. The role of hypoxia in modulating IH in the grafts has not been fully understood. Some studies have shown that hypoxia could contribute to the development of vascular pathologies like atherosclerosis and IH [10, 21]. Lee et al have shown how hypoxia at the vascular anastomosis site correlates with smooth muscle cell proliferation [11]. On similar lines, Wan et al found hypoxia and hypoxia inducible transcription factor- Hypoxia Inducible Factor 1 α (HIF1α) stabilization at the site of an arteriovenous fistula in an *in vivo* rabbit model of arteriovenous fistula [22].

We have earlier shown that hypoxia differentially modulates proliferation of vein-derived and artery-derived smooth muscle cell cultures. Vein-derived cultures proliferate much more than the artery-derived cultures, in response to VEGF-A produced by hypoxic endothelial cells [14]. Smooth muscle cell migration from the medial compartment to the intima in response to paracrine factors secreted by endothelium, smooth muscle, fibroblasts, platelets etc., is integral to the pathogenesis of IH [9]. These SMC acquire a pathological (proliferative and synthetic) phenotype in IH. Source of these abnormal SMC (host artery, circulating progenitor cells) is not completely understood [23, 24]. In the present study we investigated the differences in migration behavior between venous and arterial derived SMC in response to hypoxia. Present study shows that even basal migration of V-SMC was higher than A-SMC (Fig 1). Differences in cell migration were confirmed using three types of assays. In all of these assays, V-SMC showed higher migration. Real-time cell migration was used to determine absolute rate of cell migration. These results further confirmed that the V-SMC is more migratory than A-SMC under hypoxia.

Endothelial cell derived paracrine growth factors were found to induce SMC migration. Similar to proliferation, vein-derived cultures migrated much more than the artery-derived SMC, in response to growth factors produced by the hypoxic endothelial cells. Previously, we showed PDGF-BB is an important factor inducing proliferation of artery-derived smooth muscle cell cultures while VEGF-A was the most important factor inducing proliferation of vein-derived smooth muscle cell cultures [14]. VEGF–A is known to regulate most of its functions, including SMC proliferation through VEGFR-2, whereas SMC migration depends on VEGFR-1 [15, 25]. V-SMC migration was almost completely blocked by a neutralizing antibody against VEGFR-1 (Fig 4B). In contrast, A-SMC cells showed a different response to neutralizing antibodies. They were completely inhibited by anti-PDGD-BB antibodies whereas anti-VEGFR-1 blocking antibodies showed only a partial effect (Fig 4A). These studies demonstrate that A-SMC and V-SMC are differentially modulated by endothelial cell-derived paracrine growth factors. These observations need to be validated in *in vivo* vascular graft model systems. Further studies will address whether umbilical vein derived SMC are similar to saphenous vein derived SMC.

**Fig 7:**
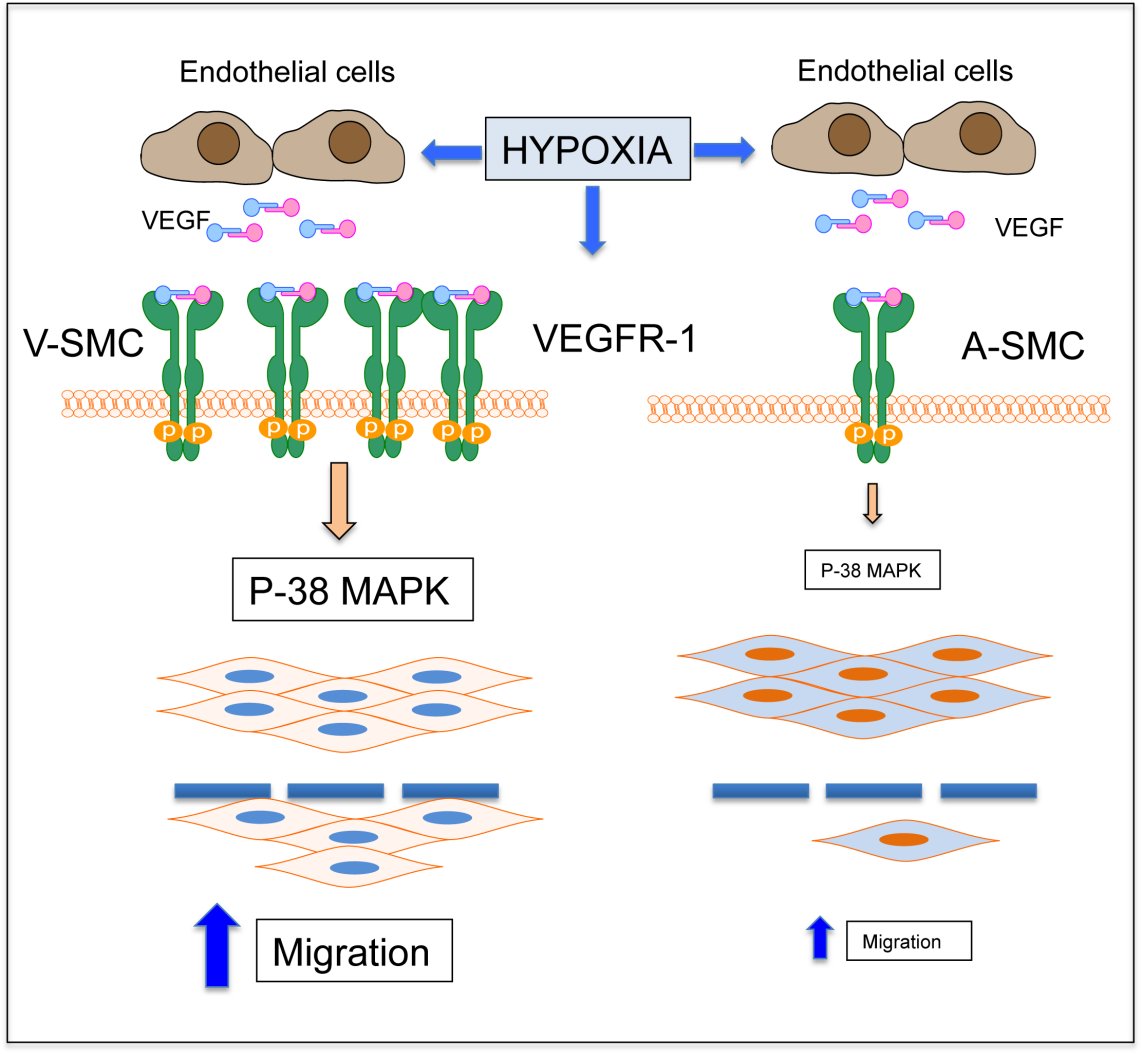
Schematic diagram showing paracrine and autocrine stimulation of SMC migration. The diagram shows a differential increase in VEGFR-1 in V-SMC and A-SMC under hypoxia, which results in phosphorylation of p38 MAPK, leading to increased migration in V-SMC compared to A-SMC.

In conclusion, we have demonstrated that hypoxia enhances SMC migration more in vein-derived SMC via VEGFR-1 dependent mechanism in contrast to A-SMC, which depend primarily on PDGF-BB. A working model is schematically shown in Fig 7. Differences in the behavior of SMC derived from a vein vs. an artery can facilitate development of new pharmacotherapy, directed at intimal hyperplasia. Such therapeutic strategies can be modulated to cater the type of vessel graft and could prevent restenosis in vascular grafts.
